# Isolation and Functional Validation of Salinity and Osmotic Stress Inducible Promoter from the Maize Type-II H^+^-Pyrophosphatase Gene by Deletion Analysis in Transgenic Tobacco Plants

**DOI:** 10.1371/journal.pone.0154041

**Published:** 2016-04-21

**Authors:** Jiajia Hou, Pingping Jiang, Shoumei Qi, Ke Zhang, Qiuxia He, Changzheng Xu, Zhaohua Ding, Kewei Zhang, Kunpeng Li

**Affiliations:** 1 The Key Laboratory of Plant Cell Engineering and Germplasm Innovation, Ministry of Education, School of Life Science, Shandong University, Shanda South Road 27, Jinan, Shandong, 250100, China; 2 Biology Institute of Shandong Academy of Sciences, Jinan, Shandong, China; 3 RCBB, College of Resources and Environment, Southwest University, Tiansheng Road 2, Beibei Dist., 400716, Chongqing, China; 4 Maize Institute of Shandong Academy of Agricultural Sciences, Jinan, Shandong, China; CSMCRI, INDIA

## Abstract

Salinity and drought severely affect both plant growth and productivity, making the isolation and characterization of salinity- or drought-inducible promoters suitable for genetic improvement of crop resistance highly desirable. In this study, a 1468-bp sequence upstream of the translation initiation codon ATG of the promoter for *ZmGAPP* (maize Type-II H^+^-pyrophosphatase gene) was cloned. Nine 5´ deletion fragments (D1–D9) of different lengths of the *ZmGAPP* promoter were fused with the GUS reporter and translocated into tobacco. The deletion analysis showed that fragments D1–D8 responded well to NaCl and PEG stresses, whereas fragment D9 and *CaMV 35S* did not. The D8 segment (219 bp; -219 to -1 bp) exhibited the highest promoter activity of all tissues, with the exception of petals among the D1–D9 transgenic tobacco, which corresponds to about 10% and 25% of *CaMV 35S* under normal and NaCl or PEG stress conditions, respectively. As such, the D8 segment may confer strong gene expression in a salinity and osmotic stress inducible manner. A 71-bp segment (-219 to -148 bp) was considered as the key region regulating *ZmGAPP* response to NaCl or PEG stress, as transient transformation assays demonstrated that the 71-bp sequence was sufficient for the salinity or osmotic stress response. These results enhance our understanding of the molecular mechanisms regulating *ZmGAPP* expression, and that the D8 promoter would be an ideal candidate for moderating expression of drought and salinity response genes in transgenic plants.

## Introduction

Water deficiency and salinity levels affect plant growth and crop yields, as they induce osmotic stress and ionic toxicity, issues that are becoming increasingly serious problems in agricultural areas worldwide [1–4]. Manipulation of plant genomes has the potential to promote revolutionary changes in crops [5], with translocated genes playing important roles in determining the phenotypic expression of transgenic plants [6]. Selection of appropriate promoters allows transgenes to be expressed at desired levels, thereby providing more precise control of transgenic plants [7–9]. The availability of various promoters that differ in their ability to regulate transgenic expression patterns can greatly facilitate the successful application of transgenic techniques [8]; as such, isolation and validation of the various promoters suitable for plant genetic transformation is therefore, necessary.

At present, the promoters used in the genetic transformation of plants are generally divided into three categories, consisting of constitutive, tissue- or stage-specific and inducible promoters. Constitutive promoters, such as the *CaMV 35S* promoter and the maize ubiquitin promoter, are widely used to direct transgene expression in almost all plant tissues at all development stages [10, 11], which frequently causes additional metabolic burden or toxic effects that result in morphological and physiological dysfunctions in plants [12, 13]. For example, both *35S*::*DREB1A* and *rd29A*::*DREB1A* transgenic tobacco plants displayed enhanced cold and drought tolerances than did control plants, but the *35S*::*DREB1A* transgenic plants experienced much more severe growth retardation than did the *rd29A*::*DREB1A* transgenic plants. The results showed that the stress-inducible *rd29A* promoter minimized negative growth effects on plant growth while conferring higher tolerance to adverse environmental conditions [14]. Indeed, the use of a tissue-specific or inducible promoter has been shown to be an ideal strategy for the elimination of negative effects resulting from constitutive overexpression of transgenes [14, 15]. Many tissue-specific (such as seed [16–19], anther [20–22], root [23–26] and green tissue-specific [27–29]) and inducible (such as drought [3, 30–32], salinity [3, 24, 30, 31, 33–35], pathogen [36] and hormone [3, 37, 38] induced) promoters have been described [6, 8, 39]. However, the majority of the reported tissue-specific or inducible promoters display weak ability in directing gene expression, which restricts their application. A shortage of available promoters with the desired expression profile limits the fine-tune control of transgene expression in plants.

H^+^-translocating pyrophosphatases (H^+^-PPase) activate proton transport across membranes by catalytic hydrolysis of inorganic pyrophosphate to provide energy [40, 41]. Higher plants have two distinct H^+^-PPase subclasses: Type I is stimulated by K^+^, whereas Type II is hypersensitive to Ca^+^ instead of K^+^ [42]. Several studies have shown that the transcriptional expression of genes encoding Type I H^+^-PPase in a number of plant species (such as *Salicornia europaea*, *Suaeda corniculata*, wheat, *Suaeda salsa*, and *Thellungiella halophila*) was induced by drought or salt stress [43–47]. Overexpression of Type I H^+^-PPase in *Arabidopsis* [44, 46, 48], tobacco [43, 45], wheat [47], maize [49], sugar beet [50], cotton [51–53], tomato [54], alfalfa [55] and creeping bentgrass [56] increased salt or drought tolerance of the transgenic plants. Sun et al. (2010) found that the *TsVP1* (Type I H^+^-PPase gene) promoter from *Thellungiella halophila* was highly active in leaves and roots, and could be induced by NaCl treatment, with a 130-bp segment identified as the key region for salt-stress response [34]. In contrast with Type I H^+^-PPase, only two Type II H^+^-PPase genes have been identified on higher plants (*AVP2* from *Arabidopsis* and *ZmGAPP* from maize). Drozdowicz et al. (2000) cloned an *Arabidopsis* Type II H^+^-PPase gene *AVP2*, the amino acid sequence of which is 36% identical to that of the Type I H^+^-PPase encoded by *AVP1* [57]. Mitsuda et al. (2001) demonstrated that AVP2 is localized primarily in the Golgi apparatus; these same authors also investigated the tissue-specific expression patterns of *AVP2* using a promoter–GUS reporter system [58], finding that *AVP2* differed from *AVP1* in that it is highly expressed in the trichome and the stamen filaments [58]. Our laboratory previously isolated the cDNA sequence of a Type II H^+^-PPase gene in maize (*ZmGAPP*; GenBank accession no. EF051578). The full-length cDNA sequence of *ZmGAPP* is 2974 bp including 2400 bp protein coding sequence, 215 bp 5' UTR and 359 bp 3' UTR [59]. Based on the length of 5' UTR, we speculated the transcription start stie of *ZmGAPP* in maize may be located in the translation initiation codon ATG upstream of approximately 215 bp. The transcription of *ZmGAPP* is enhanced in response to dehydration, cold, and salt stresses [59], but the promoter of *ZmGAPP* has thus far not been well defined. Thus, isolation and characterization of the *ZmGAPP* promoter will provide novel insights into understanding the transcriptional regulation of *ZmGAPP* and the promoter resources for plant genetic transformation.

## Materials and Methods

### Isolation of *ZmGAPP* promoter from *Zea mays* L.

The 5' flanking sequence of *ZmGAPP* was retrieved from the NCBI High Throughput Genomic Sequences Database of *Zea mays* using its full-length cDNA sequence (GenBank accession no. EF051578) as query. The forword and reverse primers (named pZmGAPPFR, Table 1) were designed according to the *ZmGAPP* sequence and its 5' flanking sequence. The 1584-bp fragment (–1468 to +116 bp; the “A” of the translation start codon “ATG” of *ZmGAPP* was designated as “+1”) was amplified from maize genomic DNA with the pZmGAPPFR primers (Table 1). The PCR conditions were as follows: initial denaturation at 95°C for 5 min followed by 35 cycles of 95°C 1 min, 55°C 1 min, and 72°C 2 min, and then final extension at 72°C for 7 min. The PCR products were excised from a 1% agarose gel and purified by AxyPrepTM DNA Gel Extraction Kit (Axygen Scientific, Inc, China). Then the fragment (–1468 to +116 bp) were cloned in the pGEM-T^®^ Easy cloning vector (Promega, USA) following the manufacturer's instructions and confirmed by sequencing. Finally, a 1468-bp fragment upstream of the translation start codon of *ZmGAPP* was isolated by PCR amplification using D1 primers (Table 1) and considered as the full-length promoter.

**Table 1:** PCR primers used in the current study.

Name	Forward (5’ to 3’)	Reverse (5’ to 3’)
**pZmGAPPFR**	cctgacttaatcgcacccat	ggagaaagattagcgaaagcc
**D1**	cccaagcttcctgacttaatcgcac	ccg*gaattc*gatggaatatgagtttg
**D2**	cccaagctttttgttgggcttagtg	ccg*gaattc*gatggaatatgagtttg
**D3**	cccaagcttgcttcgttgctgcctt	ccg*gaattc*gatggaatatgagtttg
**D4**	cccaagctttcgtgaaatcaagtgg	ccg*gaattc*gatggaatatgagtttg
**D5**	cccaagctttagaatcgctacttgc	ccg*gaattc*gatggaatatgagtttg
**D6**	cccaagcttctactgccattgtcac	ccg*gaattc*gatggaatatgagtttg
**D7**	cccaagcttagaaggtgtctgggta	ccg*gaattc*gatggaatatgagtttg
**D8**	cccaagcttgtaggcttgacggcaa	ccg*gaattc*gatggaatatgagtttg
**D9**	cccaagcttgtgtttaacttttagg	ccg*gaattc*gatggaatatgagtttg
**p35SFR**	aatggatccaagtctcaatagcccttt	tgagaattccgtattggctagagcagc
**p71bpFR**	taaggatccgtaggcttgacggca	aaactgcaggtaaacacatccaga
**HPTFR**	cgtctgctgctccatacaa	tgtcctgcgggtaaatagc
**GUSFR**	acggatggtatgtccaaagc	aacgtatccacgccgtattc
**Ntα-Tub1FR**	atgagagagtgcatatcgat	ttcactgaagaaggtgttgaa

The underlined sites are the sites for the digestion of restriction enzymes HindШ. The underlined italicized sites are the sites for the digestion of restriction enzymes EcoR1.

### Analysis of the *ZmGAPP* promoter sequence

**Fig 1:**
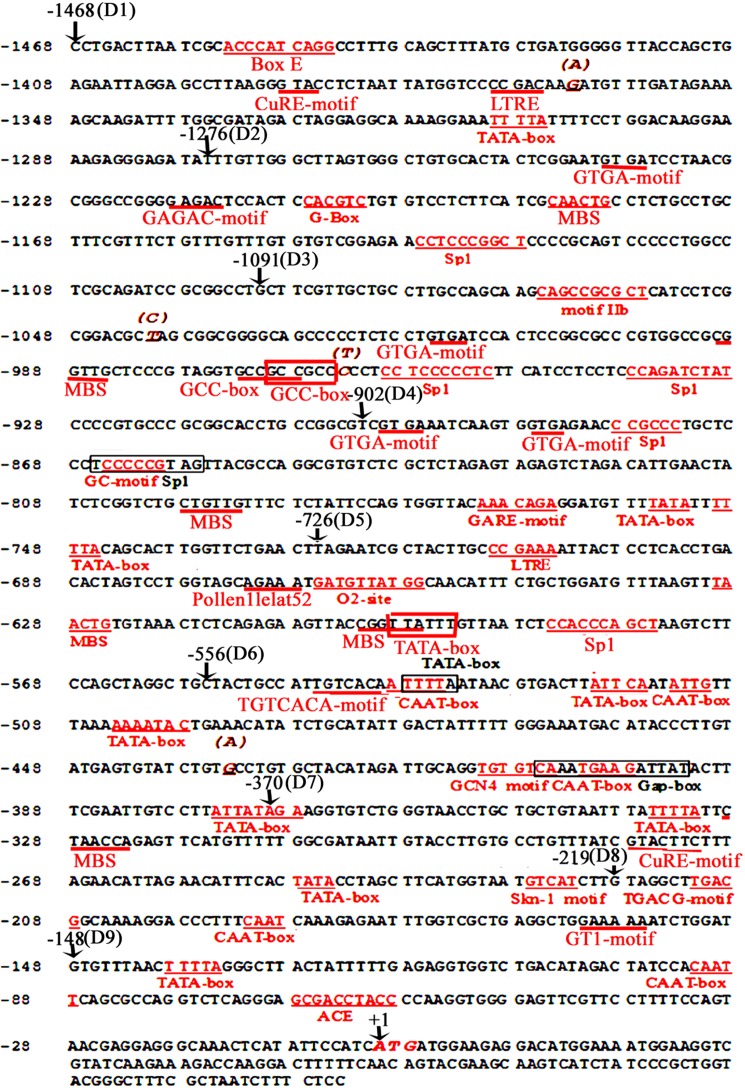
Nucleotide sequence of the *ZmGAPP* promoter. The “A” of the translation initiation code “ATG” of *ZmGAPP* was designated as “+1”. Putative *cis*-acting elements underlined or shown in the border. See Table 2 for descriptions of the elements. The arrow above the sequence indicates the start point of different deletion fragments (D1–D9).

The 1468-bp (–1468 to –1 bp) sequence of the *ZmGAPP* promoter was searched to locate the potential *cis*-acting elements using PLACE (http://www.dna.affrc.go.jp/PLACE/) and PlantCARE (http://bioinformatics.psb.ugent.be/webtools/plantcare/html/) [60, 61]. The position and description of the predicted *cis*-acting elements are listed in Fig 1 and Table 2.

**Table 2:** Identification of *cis*-acting elements in the *ZmGAPP* promoter sequence using the PLACE and PlantCARE databases.

*Cis*-elements	Description	Position from ATG	No.
**Box E**	*Cis*-acting element for induction upon fungal elicitation	-1454	1
**CuRE-motif**	Copper-response element	-1389, -278	2
**LTRE**	Low temperature responsive element	-1370, -710	2
**TATA-box**	Core promoter element around -30 of transcription start	-1310, -756, -750, -599, -538, -521, -504, -375, -336, -248, -139	11
**GTGA-motif**	*Cis*-acting element involved in late pollen development and pectate lyase	-1240, -1016, -900, -887	4
**GAGAC-motif**	Sulfur-responsive element	-1217	1
**G-Box**	*Cis*-acting element involved in light responsiveness	-1207	1
**MBS**	MYB binding site involved in drought-inducibility	-1185, -990, -798, -630, -602, -329	6
**Sp1**	Light responsive element	-1137, -960, -938, -879, -866, -585	6
**motif IIb**	Abscisic acid responsive element	-1066	1
**GCC-box**	*Cis*-acting element involved in ethylene, jasmonate and defence responsiveness	-973, -970	2
**GC-motif**	Enhancer-like element involved in anoxic specific inducibility	-865	1
**GARE-motif**	Gibberellin-responsive element	-771	1
**Pollen1lelat52**	*Cis*-acting element required for pollen specific expression	-672	1
**O2-site**	*Cis*-acting regulatory element involved in zein metabolism regulation	-666	1
**TGTCACA motif**	Enhancer element necessary for fruit-specific expression	-546	1
**CAAT-box**	Common *c*is-acting element in promoter and enhancer regions	-545, -514, -406, -192, -92	5
**GCN4-motif**	*Cis*-acting element involved in endosperm expression	-411	1
**Gap-box**	Part of a light responsive element	-404	1
**Skn-1_motif**	*Cis*-acting element required for endosperm expression	-227	1
**TGACG-motif**	*Cis*-acting element involved in the MeJA-responsiveness	-212	1
**GT-1 motif**	*Cis*-acting element involved in pathogen and NaCl induced expression	-162	1
** ACE**	*Cis*-acting element involved in light responsiveness	-66	1

### Construction of the promoter::*GUS* plasmids

For functional validation of the *ZmGAPP* promoter, nine 5′ deleted fragments (D1–D9) of different lengths (-1468 bp, -1276 bp, -1091 bp, -902 bp, -726 bp, -556 bp, -370 bp, -219 bp and -148 bp to -1 bp; Fig 1 and S1A Fig) were amplified by PCR from the 1468-bp promoter sequence of *ZmGAPP* using the primers listed in Table 1. To construct the *ZmGAPP* promoter::*GUS* plasmids, each amplified fragment was subsequently ligated into the vector pCAMBIA1391Z (Cambia, Australia) with HindШ/EcoRI restriction sites, and confirmed by restriction digestion analysis (S1B Fig) and sequencing. The resulting constructs were used for the tobacco transformation.

The minimal *CaMV 35S* promoter sequence (-46 to +10 bp) was amplified by PCR using the primer p35SFR (Table 1) and confirmed by sequencing; the fragment was then inserted into the PstI/SpeI sites upstream of the reporter gene *GUSA* in the vector pCAMBIA1304 (Cambia, Australia). The plasmid was designated as p-mini35S. The 71-bp fragment of the *ZmGAPP* promoter (-219 to -148 bp) was isolated by PCR using the primer p71bpFR (Table 1) and then confirmed by sequencing; the fragment was then inserted into the BamH1/Pst1 sites of the p-mini35S vector. The plasmid was given the name p-71bp-mini35S and used for the tobacco transient assay. The pCAMBIA1304 vector containing the *CaMV 35S* promoter upstream from the *GUSA* was used as a positive control.

### Tobacco culture and genetic transformation

Tobacco (*Nicotiana benthamiana*) seeds were sterilized with 70% ethanol for 1 min, 10% NaClO for 8 min, and then washed 5–6 times with sterile water and allowed to germinate at 25°C for 1 week. The seedlings were then transferred into culture bottles containing 50% MS nutrient medium, sucrose (30 g/L) and 0.7% agar (pH 6.0), and grown in a tissue culture chamber at 25°C under a 16-h light (220–260 μmol m^–2^ s^–1^) regime daily for 6 weeks, until transformation.

The pCAMBIA1304 and D1–D9 plasmids were transferred into *Agrobacterium tumefaciens* strain GV3101 using a freeze–thaw method. The transformation of tobacco leaf discs was performed as described by Voelker et al. (1987), with minor modifications [62]. Transformed leaf discs were screened on an MS medium supplemented with 0.1 mg/L indole-3-acetic acid, 1.0 mg/L 6-benzylaminopurine, 15 mg/L hygromycin B and 400 mg/L cefotaxime. Regenerated shoots were rooted on an MS medium containing 15 mg/L hygromycin B and 200 mg/L cefotaxime. The transformed plants were grown in soil under day/night temperatures of 25–28°C (day)/19–22°C (night) and a 16-h light (220–260 μmol m^–2^ s^–1^) cycle. The T0 transgenic plants were screened out for propagation by PCR (S2A Fig) of the hygromycin resistant gene located in the pCAMBIA1304 and pCAMBIA1391Z vectors with the primer HPTFR (Table 1) and GUS staining (S2B Fig). The transgenic tobacco lines that displayed a Mendelian segregation ratio of 3:1 in T1-generation seedlings by GUS staining were selected for subsequent propagation. Finally, three homozygous transgenic lines, each containing a single copy of the promoter::*GUS* insert from the *ZmGAPP* promoter deletion construct D1–D9 and the *CaMV 35S* promoter, were selected for subsequent function analyses using T3-generation plants.

### NaCl and PEG stress treatments

D1–D9 and *CaMV 35S* promoter transgenic and non-transgenic (WT) tobacco plants were grown under conditions of 25/19°C ± 3°C (day/night temperatures), a 16-h light (220–260 μmol m^–2^ s^–1^) cycle and approximately 65% relative humidity for 2 months, then subjected to NaCl and PEG 6000 stress treatments. Two fully expanded leaves per 60-day-old plant were used for the detached-leaves treatments. Leaf discs of 0.5 cm in diameter were cut out and floated in a liquid 1/2 MS medium supplemented with either 200 mM NaCl (salt stress treatment) or 18% (w/v) PEG 6000 (osmotic stress treatment) at 25°C for 1, 3, 6, 12, 16, 24, 48, and 72 h. The leaf discs floated in 1/2 MS liquid medium were considered the control. For whole-plant treatments, 60-day-old tobacco plants were immersed in a liquid 1/2 MS medium supplemented with either 200 mM NaCl (salt stress treatment) or 18% (w/v) PEG 6000 (osmotic stress treatment) at 25°C for 24 h. The control plants were grown in 1/2 MS liquid medium. Leaf tissues were then immediately sampled for GUS histochemical staining, and frozen in liquid nitrogen and stored at -80°C in preparation for GUS fluorometric assays. All experiments were repeated in triplicate with independent samples.

### qRT-PCR analysis

Total RNA was isolated from leaves of transgenic tobacco plants using the TRIzol reagent (Sangon, China) and then treated with RNase-free DNase (Takara, China). The cDNA synthesis was performed with the RT reagent kit (Takara, China) according to the manufacturer’s protocol. The qRT-PCR assays were performed using the SYBR Green RT-PCR Kit (Takara, China) on a Chromo^TM^ 4 Gene Amplification System (MJ Research, USA), in a 10 μl reaction volume containing 5 μL of SYBR Green PCR mix, 0.2 μM of each forward and reverse primer, 1 μL of diluted cDNA template, and the appropriate amount of sterile ddH_2_O. The amplification conditions were as follows: 2 min at 95°C, 40 cycles of 15 s at 95°C, 30 s at 58°C, and 30 s at 72°C. The relative expression level of RNA transcripts were calculated by the 2^-ΔΔCt^ method [63]. As the expression of tobacco α-tubulin (Ntα-Tub1; AJ421411) is known to be fairly uniform; it was used as an internal control to normalize the expression of *GUS*. The entire experiment was repeated three times with independent samples, and the primer sequences (GUSFR and Ntα-Tub1FR) are shown in Table 1.

### GUS histochemical and fluorometric analysis

GUS histochemical staining and fluorometric assay were performed according to the methods described by Jefferson et al. (1987) with minor modifications [64]. The tissues were placed in GUS staining solution containing 50 mM sodium phosphate (pH 7.0), 0.5 mM potassium ferricyanide, 0.5 mM potassium ferrocyanide, 10 mM EDTA, 0.1% Triton X-100 and 1 mM X-Gluc (Sangon, Shanghai, China), then D1-D3 and D4-D9 fragments incubated at 37°C for 24 h and 6 h, respectively. Because of the original high GUS expression levels in D4-D9 transgenic tobacco plants, they require shorter incubation time in order to better evaluate intensity of the reaction. To make clear the difference of GUS staining between D4-D9 before and after stresses, GUS staining for 6 h was determined by the pre experiment. After staining, the tissues were bleached with 70% ethanol and photographed (Sony DSC-F828 digital camera).

Leaf tissues were homogenized in a 4°C extraction buffer containing 50 mM sodium phosphate (pH 7.0), 0.1% sodium lauryl sarcosine, 10 mM DTT, 0.1% Triton X-100 and 10 mM EDTA for GUS fluorometric assays. The samples were centrifuged for 15 min at 10000 g and 4°C, with supernatant activity detected via an assay buffer containing 1 mM 4-methylumbelliferyl-b-glucuronide (4-MUG, Sigma, USA) at 37°C. The reaction was terminated by the addition of 200 mM Na_2_CO_3_, to a final concentration of 180 mM. Fluorescence was measured with a fluorescence spectrophotometer (HITACHI F-4600, Japan) at the excitation and emission wavelengths of 365 nm and 455 nm, respectively. Protein concentration of the supernatant was determined using the Bradford method [65]. The GUS activity was calculated as nmol of 4-Methylumbelliferone (4-MU) per mg protein per minute under controlled conditions.

### GUS transient expression assay

Transient expression of GUS activity was carried out using leaves of 60-day-old tobacco plants as described previously [66]. *Agrobacterium tumefaciens* GV3101 harboring p-mini35S, p-71bp-mini35S and pCAMBIA1304 plasmids were grown on YEP medium containing 50 mg/L Rif and 50 mg/L kanamycin at 28°C for 18 h. The *Agrobacterium* cultures were isolated by centrifugation for 15 min at 6000 g, resuspended in the infiltration medium containing 10 mm MES, 100 μm acetosyringone and 10 mm MgCl_2_ (pH 5.6) to an OD_600_ of 0.6, and incubated at room temperature for 3 h. The *Agrobacterium* cultures were then agro-injected into tobacco leaves at the abaxial surfaces using a needleless syringe, following which the agro-infiltrated plants were maintained in a moist chamber at 25°C for 48 h.

For NaCl and PEG stress treatments, the infiltrated leaf discs were cut out and floated on a liquid 1/2 MS medium supplemented with either 200 mM NaCl (salt stress treatment) or 18% (w/v) PEG 6000 (osmotic stress treatment) for 24 h. The infiltrated leaves incubated in the liquid 1/2 MS medium were considered the control. Leaf tissues from fifteen independently infiltrated plants were then used for GUS histochemical staining and GUS fluorometric assays. All experiments were repeated in triplicate.

### Data analysis

Results were expressed as mean values ± SD (standard deviation). A Student’s t test (n = 3, P < 0.05; Sigmaplot 12.0) at a 95% confidence level was used to test for statistical significance.

## Results

### Isolation of *ZmGAPP* promoter from *Zea mays* L. and sequence analysis

Based on the public sequence from MaizeGDB (http://www.maizegdb.org/), the 1468-bp 5′ flanking sequence of *ZmGAPP* upstream of the start codon ATG was obtained from maize genomic DNA. The *ZmGAPP* promoter sequence was analyzed using the online software PlantCARE and PLACE. Twenty-three kinds of potential c*is*-acting elements were present in the 1468-bp region of the *ZmGAPP* promoter (Fig 1 and Table 2). Multiple core *cis*-acting elements, including 11 TATA and 5 CAAT boxes, were found at numerous positions. A series of putative *cis*-regulatory elements that enables the inducible or tissue-specific expression of *ZmGAPP* were identified, including four types of light-responsive elements (G-Box, Sp1, Gap-box and ACE), four kinds of hormone-responsive elements (motif IIb, GCC-box, GARE motif and TGACG motif), a copper-responsive element (CuRE motif), a sulfur-responsive element (GAGAC-motif), a *cis*-acting element involved in pathogen- and NaCl-induced expression (GT1), two low-temperature-responsive elements (LTRE), a fungal-inducible element (Box E), six MYB binding sites involved in drought-inducibility (MBS), an enhancer-like element involved in anoxic specific inducibility (GC motif), a zein-metabolism-related element (O2-site) and several elements required for tissue-specific expression (GTGA motif, Pollen1lelat52, TGTCACA motif, GCN4 motif and Skn-1 motif).

### Expression patterns and activities of *ZmGAPP* promoter and its 5′ deletion segments in transgenic tobacco plants under normal conditions

**Fig 2:**
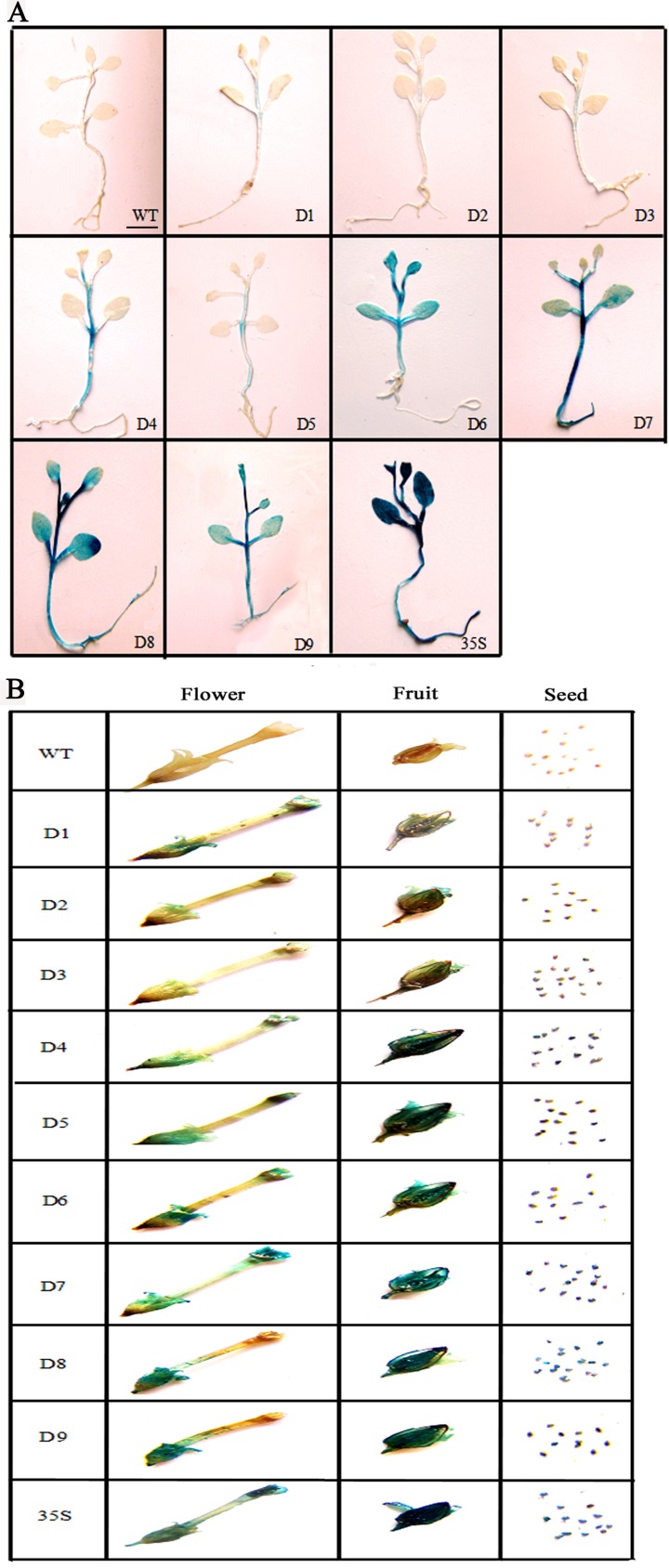
GUS histochemical assays of tissues of D1–D9 and *CaMV 35S* transgenic tobacco plants. Twenty-day-old seedlings (A), and flowers, fruits and seeds (B) were incubated in staining solution at 37°C. The D1–D3 and D4–D9 fragments were stained for 24 h and 6 h, respectively, following which the samples were observed and photographed after decolorization. Scale bar: 0.5 cm.

Our results suggest that all nine of the *ZmGAPP* promoter deletion segments (D1–D9) could direct GUS expression in transgenic tobacco, but they differed considerably in expression patterns and activities. To assess the expression patterns of both D1–D9 and the *CaMV 35S* promoter under normal conditions, the flowers, fruits, seeds, and 20-day-old seedlings of transgenic tobacco were tested via GUS histochemical staining (Fig 2).

For 20-day-old seedlings (Fig 2A), weak GUS expression was detected in the stems of D1–D3 and D5, whereas GUS expression was virtually absent in the roots, cotyledons and leaves. GUS expression in the D4 seedlings was also detected only in the stems, and GUS-expression intensity was stronger than that in D1–D3 and D5 transgenic tobacco. GUS was strongly expressed in all tissues of D6–D9 seedlings, with the exception of the roots of D6 mutants. The deletion of the 912-bp (–1468 to –556 bp) promoter fragment located between D1 and D6 enhanced GUS activity in the leaves and cotyledons of the seedlings, suggesting that the 170-bp (–726 to –556 bp) segment between D5 and D6 may contain the *cis*-acting elements that inhibited gene expression in the leaves and cotyledons of 20-day-old tobacco seedlings. Three TATA boxes, a pollen-specific expression required element (Pollen1lelat52) and several elements responsive to low temperatures (LTRE), zein metabolism (O2-site), drought (MBS), light (Sp1) and gibberellin (GARE motif) were present in the 170-bp region (Fig 1 and Table 2). Given that no expected elements were detected, the 170-bp sequence may thus contain unknown elements that inhibit gene expression in the leaves and cotyledons of 20-day-old tobacco seedlings. Moreover, GUS expression was absent in the roots of D1–D6 seedlings, whereas strong GUS expression was detected in the roots of D7–D9 tobacco seedlings. We would expect *cis*-acting elements that inhibit gene expression in the roots of 20-day-old tobacco seedlings to be present in the 186-bp (–556 to –370 bp) region between D6 and D7, yet only a fruit-specific expression required element (TGTCACA motif), a GCN4 motif involved in endosperm expression, a light-responsive element (Gap box), three CAAT boxes and two TATA boxes were found in this sequence. Therefore, the 186-bp sequence may contain no reported elements that inhibit gene expression in the roots of 20-day-old tobacco seedlings.

For flowers, fruits and seeds (Fig 2B), GUS expression could be detected in the transgenic tobacco plants with the *ZmGAPP* promoter and its 5′ deletion segments, with the exception of the petals of D8 and D9, and the GUS-expression intensity of D1–D9 was similar to that in the 20-day-old tobacco seedlings. GUS expression activities in D1–D3 transgenic tobacco were weak in the flowers, fruits and seeds, whereas, with the exception of the petals of D8 and D9, GUS expression was stronger in D4–D9 than that in D1–D3. Notably, the deletion of a 151-bp (–370 to –219 bp) segment between D7 and D8 resulted in the loss of GUS expression capability in the petals of D8 and D9, implying that the 151-bp (–370 to –219 bp) segment may contain some *cis*-regulatory elements required for petal-specific expression. However, only two TATA boxes, an endosperm-specific expression required element (Skn-1 motif), a copper-responsive element (CuRE motif), and a MYB binding site involved in drought inducibility (MBS) were identified in the region. As such, the 151-bp (–370 to –219 bp) segment may therefore, contain no reported elements required for petal-specific expression.

In regard to controls, GUS expression analysis of WT (negative control) and *CaMV 35S* (positive control) were also carried out (Fig 2). GUS expression of WT was not detected in all tissues of 20-day-old seedlings, flowers, fruits and seeds, whereas the *CaMV 35S* transgenic plants displayed the highest GUS-expression intensity among the tested constructs, and GUS was expressed in various tissues.

**Fig 3:**
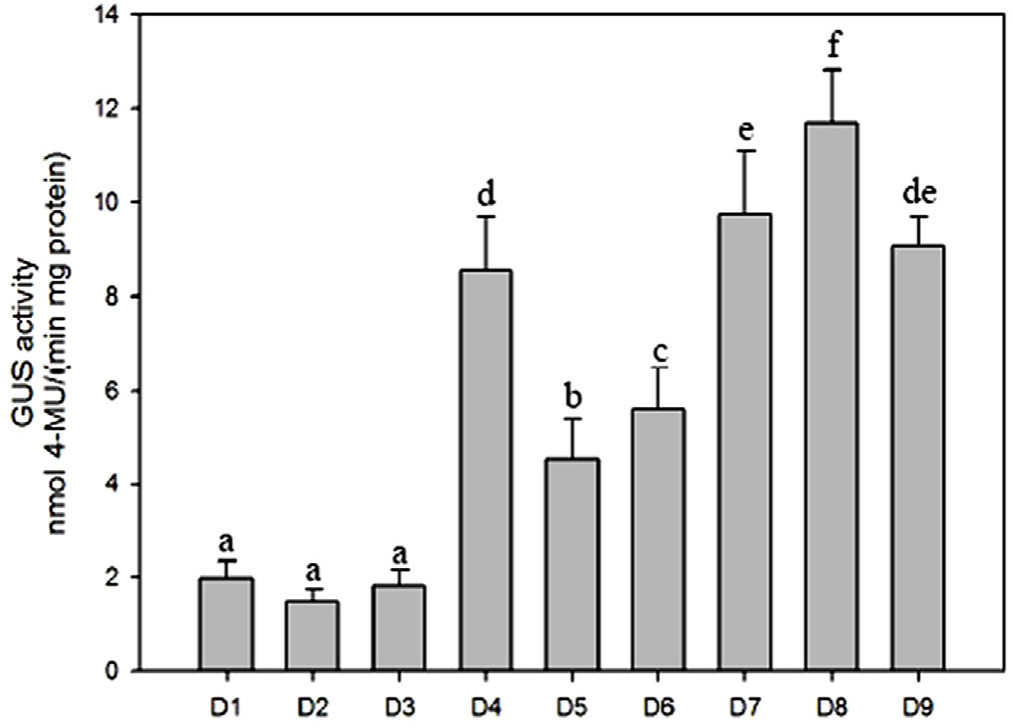
GUS activity assays of D1–D9 transgenic tobacco plants under normal conditions. Values are means ± SD from 15 independent transgenic plants (5 individual plants/ line, 3 lines for each construct). Different lowercase letters above the bars indicate significant differences at *P* < 0.05.

To further evaluate the contribution of different fragments of the *ZmGAPP* promoter to its expression activity under normal conditions and identify the core functional region, fluorometric GUS assays were performed on D1–D9 transgenic tobacco leaves of 60-day-old mature plants (Fig 3). The promoter activities of D1–D3 and D5 were relatively weak, whereas GUS expression was strong in D4 and D6–D9. The promoter activity of D4 was approximately 4.5-fold as high as that of D1–D3. A motif IIb (abscisic acid responsive element), a GTGA motif (late pollen development related element), a MBS (MYB binding site involved in drought-inducibility), two Sp1 light-responsive elements and two GCC boxes (*Cis*-acting element involved in ethylene, jasmonate and defense responsiveness) were found in the 189-bp (–1091 to –903 bp) region between D3 and D4 (Fig 1 and Table 2) by bioinformatics analysis. The results suggested that the 189-bp sequence may contain novel elements that inhibit *ZmGAPP* transcription. Moreover, GUS-expression intensity driven by D8 was considerably higher than that induced by D1–D7 and D9, which corresponds to approximately 6-fold of the full-length promoter (D1). The 219-bp (–219 to –1 bp) D8 segment may be the key sequence required for high-level expression of *ZmGAPP*, which contains the promoter core *cis*-acting elements CAAT and TATA boxes (Fig 1).

### Salinity and osmotic stress-induced activity analysis of the *ZmGAPP* promoter and its 5′ deletion segments in transgenic tobacco

To understand the molecular basis of NaCl- or PEG-inducible expression of *ZmGAPP*, the promoter activities of D1–D9 were tested in leaves by incubating the detached leaves or whole plants in liquid 1/2 MS medium supplemented with 200 mM NaCl (salt stress treatment) or 18% PEG 6000 (osmotic stress treatment). *CaMV 35S* promoter transgenic tobacco (positive control) and wild type (negative control) plants were also treated in parallel.

**Fig 4:**
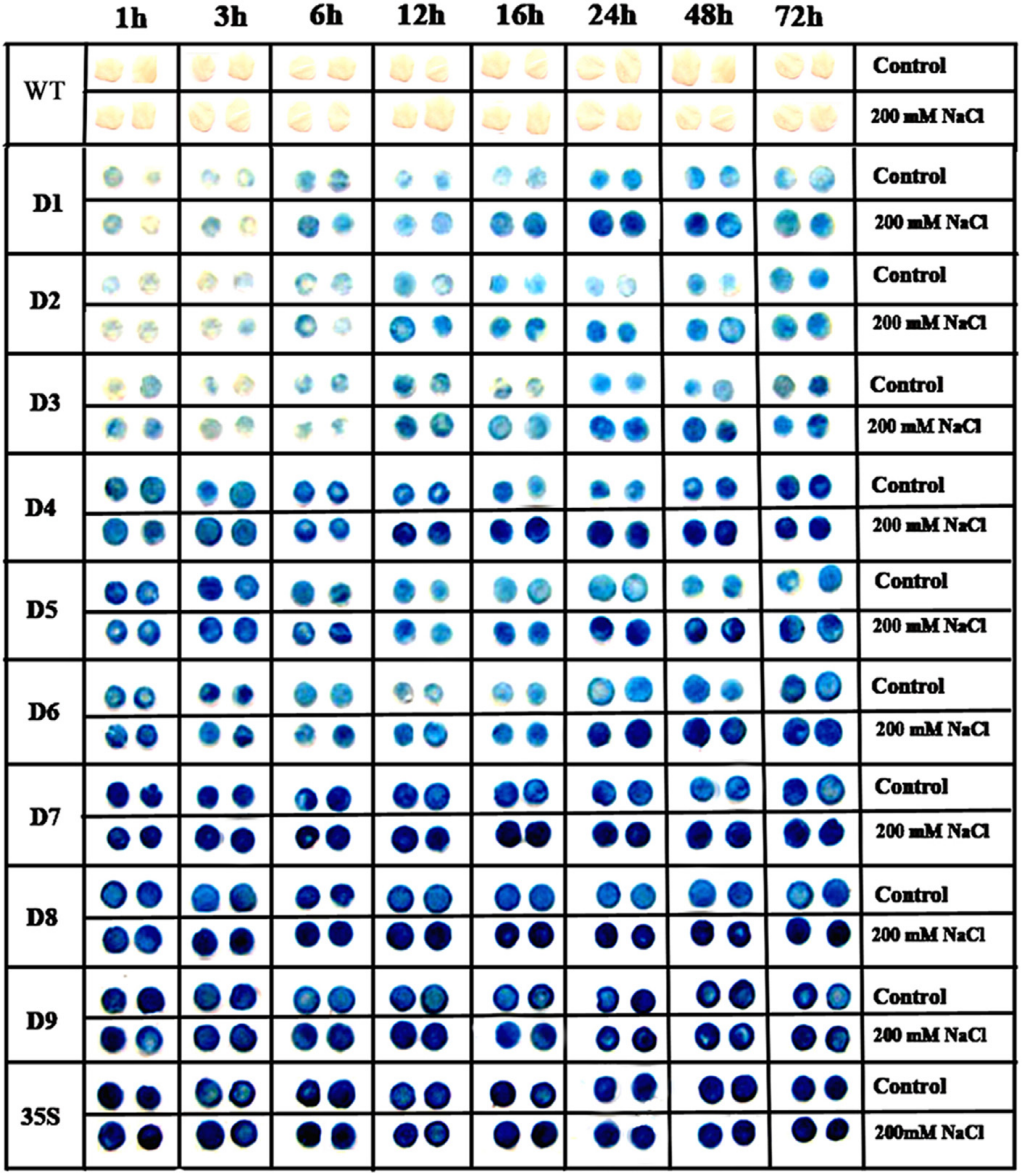
GUS staining of detached leaves of transgenic tobacco under normal and salt-stress conditions. Ninety leaf discs (diameter 0.5 cm) from 15 individual plants (5 individual plants/ line, 3 lines for each construct) of D1–D9 and *CaMV 35S* transgenic tobacco plants were incubated in liquid 1/2 MS medium supplemented with 200 mM NaCl for 1, 3, 6, 12, 16, 24, 48, and 72 h; leaf discs floated in liquid 1/2 MS medium were used as control. The leaf discs of D1–D3 plants were then incubated in staining solution at 37°C for 24 h, whereas the leaf discs of D4–D9 and *CaMV 35S* transgenic plants were stained for 6 h. Finally, the samples were observed and photographed after decolorization.

**Fig 5:**
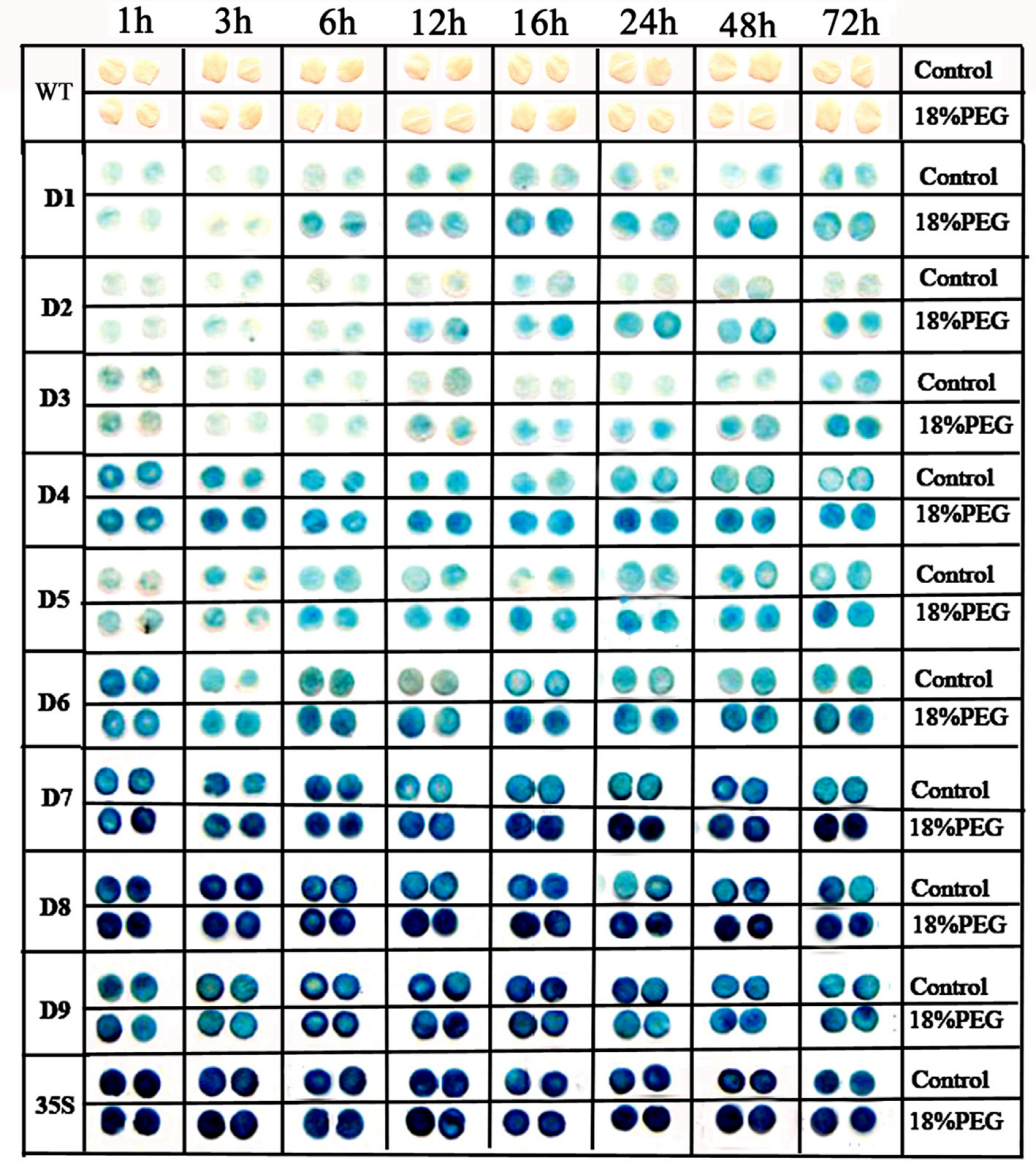
GUS staining of detached leaves of transgenic tobacco under normal and PEG treatment conditions. Ninety leaf discs (diameter 0.5 cm) from 15 individual plants (5 individual plants/ line, 3 lines for each construct) of D1–D9 and *CaMV 35S* transgenic tobacco plants were incubated in liquid 1/2 MS medium supplemented with 18% PEG 6000 (w/v) for 1, 3, 6, 12, 16, 24, 48, and 72 h; leaf discs floated in liquid 1/2 MS medium were used as control. The leaf discs of D1–D3 plants were then incubated in staining solution at 37°C for 24 h. The leaf discs of D4–D9 and *CaMV 35S* transgenic plants were stained for 6 h. Finally, the samples were observed and photographed after decolorization.

The detached leaves from 60-day-old mature tobacco plants were subjected to either 200 mM NaCl or 18% PEG 6000 treatment in a time-course experiment (Figs 4 and 5). GUS staining intensity of the leaf discs revealed no obvious differences between the stress-treated groups and control groups for 1-, 3-, or 6-h NaCl or PEG treatment. The leaf discs of D1–D8 plants displayed a stress-induced tendency following 12-h treatment, and the GUS expression level of D1–D8 leaf discs were clearly higher than those of the untreated control groups after 16-, 24-, 48-, and 72-h NaCl or PEG treatments. However, the levels of GUS expression in the leaf discs from *CaMV 35S* and D9 transgenic tobacco were stable during the NaCl and PEG treatments.

**Fig 6:**
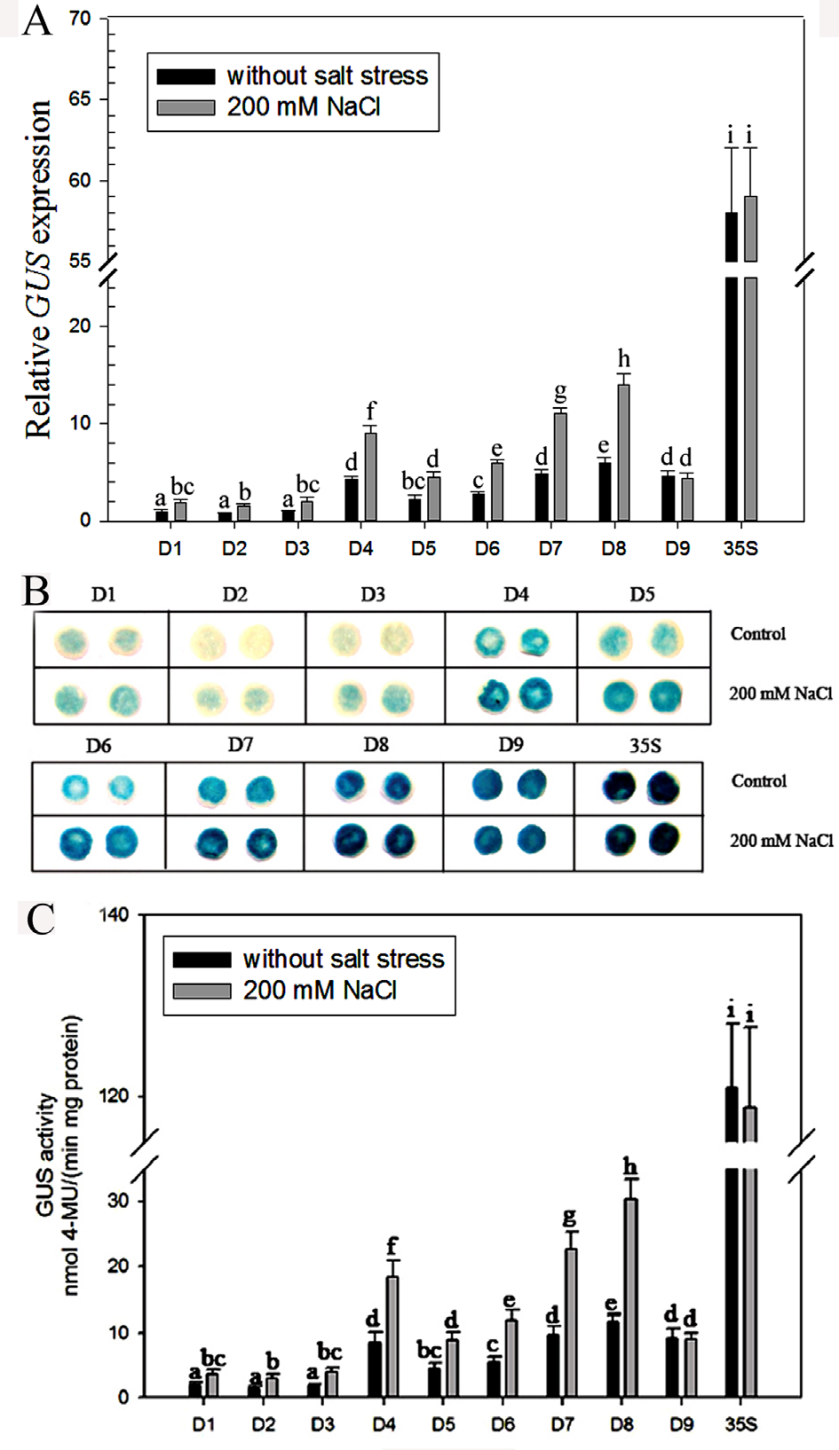
Analysis of different *ZmGAPP* promoter deletion constructs in transgenic tobacco plants under normal and NaCl treatment conditions. The D1–D9 and *CaMV 35S* transgenic tobacco plants were incubated in liquid 1/2 MS medium supplemented with 200 mM NaCl for 24 h; plants grown in liquid 1/2 MS medium were treated as control. (A) qRT-PCR analysis. The tobacco *α-tubulin* (AJ421411) was used as an internal control. (B) GUS histochemical staining. The leaves of D1–D3 plants were incubated in staining solution at 37°C for 24 h; leaves of D4–D9 and *CaMV 35S* transgenic plants were stained for 6 h. Samples were then observed and photographed after decolorization. (C) GUS activity assays. Values represent the means ± SD from 15 independent transgenic plants (5 individual plants/ line, 3 lines for each construct). Different lowercase letters above the bars indicate significant differences at *P* < 0.05.

**Fig 7:**
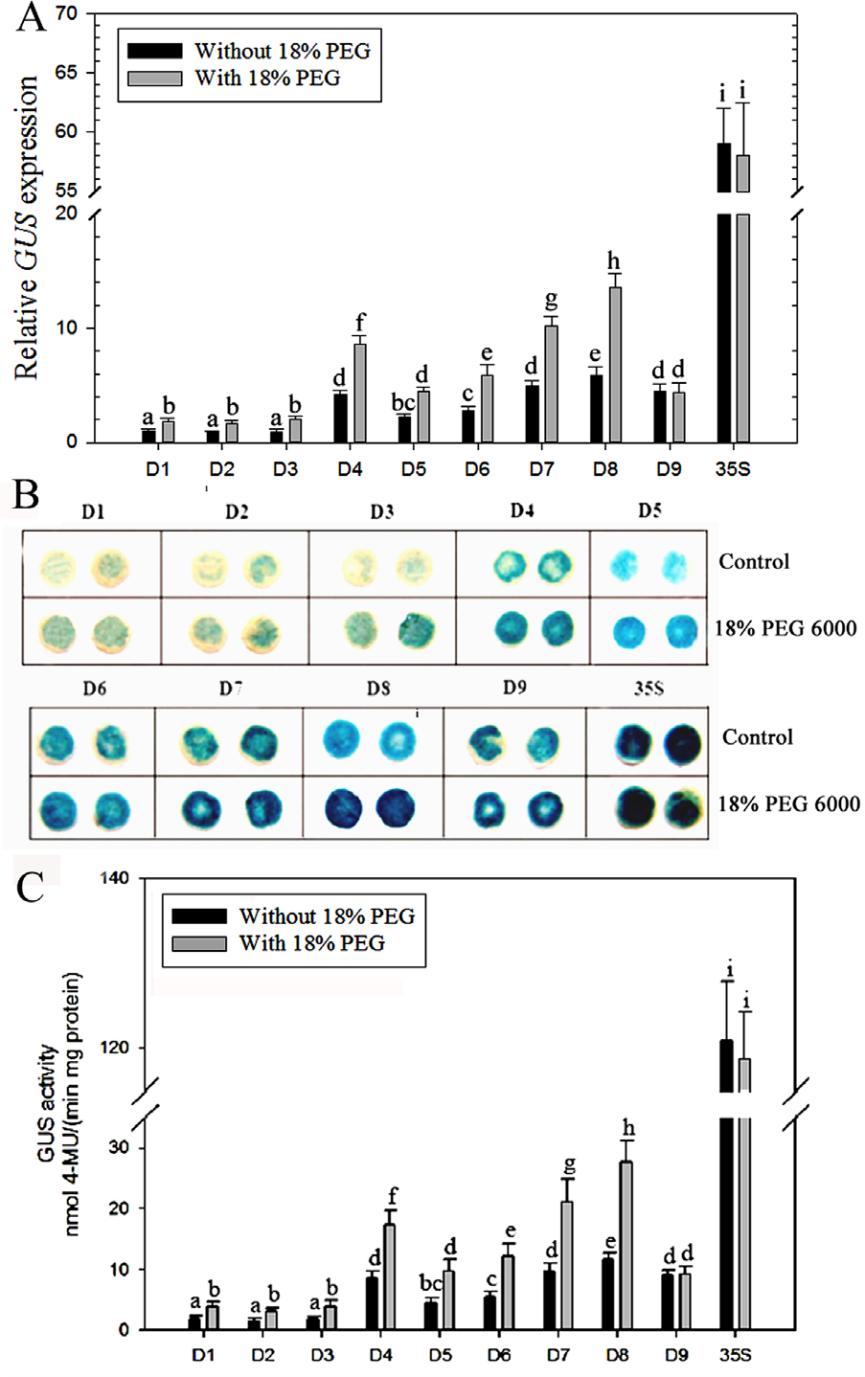
Analysis of different *ZmGAPP* promoter deletion constructs in transgenic tobacco plants under normal and PEG treatment conditions. The D1–D9 and *CaMV 35S* transgenic tobacco plants were incubated in liquid 1/2 MS medium supplemented with 18% PEG 6000 (w/v) for 24 h; plants grown in liquid 1/2 MS medium were treated as control. (A) qRT-PCR analysis. The tobacco *α-tubulin* (AJ421411) was used as an internal control. (B) GUS histochemical staining. The leaves of D1–D3 plants were incubated in staining solution at 37°C for 24 h; leaves of D4–D9 and *CaMV 35S* transgenic plants were stained for 6 h. Samples were then observed and photographed after decolorization. (C) GUS activity assays. Values represent the means ± SD from 15 independent transgenic plants (5 individual plants/ line, 3 lines for each construct). Different lowercase letters above the bars indicate significant differences at *P* < 0.05.

To further evaluate the results, stress treatments involving whole plants were also conducted. Based on the results of the detached-leaves experiment, we chose 200 mM NaCl or 18% PEG 6000 treatments for 24 h. Analysis of *GUS* expression, GUS staining intensity and enzyme activity demonstrated that the promoter activities of D1–D8 were induced by up to more than two-fold in the leaves for 24 h of 200 mM NaCl or 18% PEG 6000 treatment (Figs 6 and 7), whereas no significant differences were detected in D9 and *CaMV 35S* transgenic tobacco leaves before and after stress treatments. In other words, the D1–D8 promoter sequence appears to respond well to salinity and osmotic stress, whereas D9 and *CaMV 35S* did not, indicating that the 71-bp (–219 to –148 bp) fragment of the *ZmGAPP* promoter between D8 and D9 may contain *cis*-acting elements responsive to salinity and osmotic stress. Notably, the D8 fragment still exhibited the highest level of promoter activity among the D1–D9 under NaCl or PEG stress (Figs 6 and 7), and was fully six times more active than was the full-length promoter (i.e., D1). Under normal conditions, the promoter activity of D8 was about 1.4-fold that of D9 and 10% of *CaMV 35S*, but three-fold more so than D9 and about 25% of the *CaMV 35S* promoter after 200 mM NaCl or 18% PEG 6000 treatment for 24 h. Therefore, the D8 segment (219 bp; –219 to –1 bp) may confer high levels of gene expression and contain elements of an NaCl- or PEG-inducible nature.

### The 71-bp fragment (–219 to –148 bp) is the key region of the *ZmGAPP* promoter in terms of response to salinity and osmotic stress

**Fig 8:**
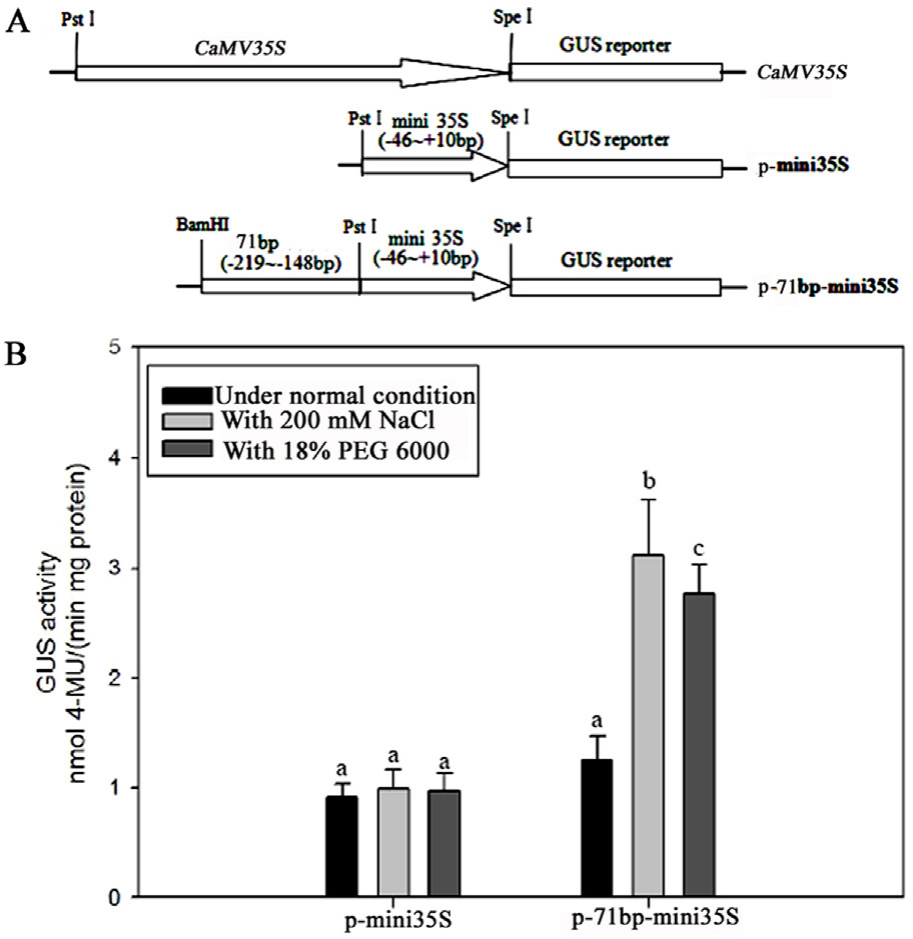
GUS transient assays in tobacco leaves. (A) The plasmids used in the transient assay. The *CaMV 35S* represents the full-length 35S promoter; p-mini35S represents the truncated 35S (–46 to +10 bp) promoter. The test construct consisted of the p-71bp-mini35S, in which the 71-bp region (–219 to –148 bp) identified in the *ZmGAPP* promoter was fused to the p-mini35S promoter to drive the GUS expression. (B) GUS activity in the transiently transformed tobacco leaves with constructs p-mini35S and p-71bp-mini35S under both normal and 200 mM NaCl or 18% (w/v) PEG 6000 treatment for 24 h. Results are mean ± SD from three experiments (n = 15). Different lowercase letters above the bars indicate significant differences at *P* < 0.05.

The 71-bp (–219 to –148 bp) segment between D8 and D9 was isolated and inserted into the p-mini35S vector, as described in the Materials and Methods section. The vector was given the name p-71bp-mini35S and used for *Agrobacterium*-mediated *GUS* transient assay in tobacco leaves in order to test its NaCl- or PEG-inducible activity (Fig 8 and S3 Fig). The GUS-expression intensity of the p-71bp-mini35S vector in transiently transfected tobacco leaves had a significant increment after 200 mM NaCl or 18% PEG 6000 treatment for 24 h, whereas the level of GUS expression in the p-mini35S promoter-transformed tobacco leaves remained largely unchanged following NaCl or PEG stress treatments (Fig 8 and S3 Fig). These results suggest that the 71-bp (–219 to –148 bp) segment contains elements of salinity and osmotic stress responsiveness.

The WT and *CaMV 35S* promoter-infiltrated tobacco leaves were also used as negative and positive controls; the *CaMV 35S* promoter-infiltrated tobacco leaves displayed strong GUS expression, whereas no GUS activity was detected in the WT tobacco leaves (S3 Fig).

## Discussion

Plants have two phylogenetically distinct subclasses of H^+^-PPase. The genes encoding Type I H^+^-PPase have been characterized in several plant species. Its transcriptional expression was up-regulated by salinity and drought [43–47]. The *TsVP1* (Type I H^+^-PPase gene) promoter from *Thellungiella halophila* displayed strong activity and NaCl stress inducibility [34]. Genetic manipulation of Type I H^+^-PPase in plants results in enhanced salinity or drought tolerance [43–56]. However, the genes encoding Type II H^+^-PPase have thus far been rarely examined. In previous studies, the ZmGAPP (Type II H^+^-PPase; GenBank accession no. EF051578) gene was cloned by our laboratory from maize, which shares only 39% of its amino acid sequence identity with that of maize VPP1 (Type I H^+^-PPase; GenBank accession no. AJ715528). The qRT-PCR analysis of *ZmGAPP* showed that its expression is up-regulated in multiple stress conditions, such as 200 mM NaCl and 18% PEG 6000 [59]. Bioinformatic analysis demonstrated that the upstream regulatory region of *ZmGAPP* contains potential *cis*-acting elements related to abiotic stresses, including salinity (GT1 motif) and drought (MBS), which may be involved in the induced expression of *ZmGAPP* (Fig 1 and Table 2). To understand the molecular basis of the stress response and identify ideal candidate promoters for the transgenic breeding of crop drought or salinity resistance, the *ZmGAPP* promoter was cloned, characterized, and functionally validated in this study.

The analysis of 5′ deleted mutants of the *ZmGAPP* promoter (D1–D9) under NaCl and PEG 6000 treatments revealed that a 71-bp sequence (–219 to –148 bp, the upstream of the translation initiation codon ATG) is the key region for *ZmGAPP* response to NaCl or PEG stress. GUS transient assay of leaves of 60-day-old tobacco plants displayed that this 71-bp sequence was sufficient for the response of NaCl or PEG stress. Bioinformatics analysis determined that the 71-bp (–219 to –148 bp) region contains a CAAT box, a TGACG motif and a GT1 motif (GAAAAA). The TGACG motif is a *cis*-acting element involved in MeJA-responsiveness, whereas the CAAT box is a common *cis*-acting element in promoter and enhancer regions that typically exhibits a putative effect in enhancing gene expression, resulting in the increment of p-71bp-mini35S promoter activity compared with p-mini35S under normal conditions (Fig 8B). Park *et al*. (2004) found that the transcription of *SCaM-4* is dramatically induced by NaCl or pathogen treatment [67]. A GT-1 motif (GAAAAA) was ultimately identified as a core element responsible for the NaCl- or pathogen-induced expression of *SCaM-4* in part by GT-1 interaction with AtGT-3b (an Arabidopsis GT-1-like transcription factor) in both soybean and Arabidopsis [67]. In our present study, a GT-1 motif (GAAAAA) also was identified within the *ZmGAPP* promoter between -219 and -148 bp. And the 71-bp (–219 to –148 bp) sequence has been shown to respond well to salt and osmotic stresses. Further detection whether GT-1 or other motif that have not been reported involved in stress inducible response of the 71-bp fragment and identification its interacting protein will provide a better understanding on the inducible gene expression of *ZmGAPP* during salt or osmotic stress.

Moreover, the deletion of the 189-bp (–1091 to –903 bp) sequence between D3 and D4 resulted in significant increase of GUS activity in leaves of transgenic tobacco plants (Fig 3). The results showed that the 189-bp sequence mediates transcriptional repression and contributes to relatively weak promoter activity of D1-D3 fragments in tobacco leaves. However, the 189-bp fragment does not appear to contain known *cis*-acting elements that inhibit gene expression by sequence analysis. Indeed, the silencers are generally varying in size and showing sequence degeneracy that make it difficult to recognize them in comparative analysis [8]. The activities of some well-studied plant silencer are associated with tissue specific expression, regulation by light, etc. Castresana *et al*. (1988) reported a A/T-rich DNA sequence that reduced the expression of photoregulated gene *CAB* in light in *Nicotiana plumbaginifolia* [68]. Delaney *et al*. (2007) identified an 84-bp A/T-rich sequence in the cotton *FSltp4* promoter that suppressed the expression of *FSltp4* in non-fiber tissues [69]. Lai *et al*. (2009) identified a 43-bp A/T-rich element in the *AtKP1* promoter that mediated the transcriptional repression in both roots and leaves [70]. These A/T-rich DNA sequences usually mediate gene expression in plants [71], while no A/T-rich similar sequence is present in the 189-bp fragment. Further discovering the negatively regulatory element that located within the 189-bp (–1091 to –903 bp) sequence by deletion analysis and site-specific sequence mutation will promote our understanding on the transcriptional regulation of *ZmGAPP*.

Transgenic technology offers a powerful tool for gene function characterization and crop improvement [7], and appropriate promoter selection has become increasingly important for the successful application of transgenic technology [8]. Each additional transgene requires its own promoter, making it necessary to identify different promoters that achieve the same expression profile [6]. Cloning and identification of inducible or tissue-specific promoters would be of great practical value, as doing so would eliminate unnecessary burdens by restricting genetic expression to specific tissues or in response to specific environmental conditions [8, 72–74]. As such, the cloning and functional validation of salinity or drought-stress inducible promoters are therefore, of great importance to the effective management of salinity tolerance or drought resistance in commercial crops. In the current study, a 219-bp (D8) NaCl- and PEG-stress inducible core fragment extracted from the *ZmGAPP* regulatory region was identified by 5′ deleted mutant analysis. The GUS expression of D8 was highest in all tissues, with the exception of petals, among D1–D9 transgenic tobacco plants, which corresponds to about 10% and 25% of the *CaMV 35S* promoter under normal and NaCl- or PEG-stress conditions, respectively. The D8 fragment exhibited high promoter activity, especially under salt or osmotic stress, but was lower than that of the *CaMV 35S* promoter. It has been shown that excessive expression of transgenes in host plants may inhibit their growth and development, often resulting in host-plant morphological and physiological dysfunction [7, 8, 75]. Thus, the D8 fragment may be useful for moderating expression of transgenes and, more importantly, facilitates the expression of transgenes at desired levels under conditions of salt and osmotic stress.

Abiotic stress adaptability of crops is complex, and single transgene introductions may not be sufficient to improve crop stress resistance under natural conditions. Multiple-gene transformation is becoming routine in the genetic engineering of plants, as researchers strive for transgenic plants that present more complex and ambitious phenotypes [6]. However, expression of the multiple transgenes that are introduced into the host plants are regulated by the same promoter in the vector, which often results in homology dependent gene silencing [7, 76–78]. The D8 fragment is only 219 bp; such a small inducible promoter would be very useful in avoiding the repetitive usage of the same constitutive promoter and would reduce the vector size for plant genetic transformation [7], but also expresses target transgenes in an inducible manner, which will help to improve the adaptability of crops to adverse environmental conditions. It is also known that utilizing heterologous promoters to drive the expression of transgenes in host plants can help to prevent homology dependent gene silencing [79, 80]. Thus, this truncated 219-bp fragment (D8) of the maize promoter *ZmGAPP* could be used to confer high levels of gene expression and salinity or osmotic stress inducibility to transgenic tobacco plants, and as such, this monocot promoter fragment may be an ideal candidate for improving salinity or drought resistance in dicot crops. The transcriptional behave of promoters may have obvious difference in monocot and dicot. For example, *CaMV 35S* promoter displays strong transcriptional activity in dicot, while ubiquitin promoters are generally more capable of driving transgene expression in monocot [9, 81]. However, some promoters, such as 0.3 kb *AtTCTP* promoter [7], have strong transcriptional activity in both monocot and dicot. The D8 fragment derived from maize (monocot) displays high transcriptional activity in salinity and osmotic stresses inducible manner in tobacco (dicot). Further characterization of D8 promoter in monocot and evaluation of its application prospect in transgenic breeding of monocot crops will also be meaningful.

## Conclusions

**Fig 9:**
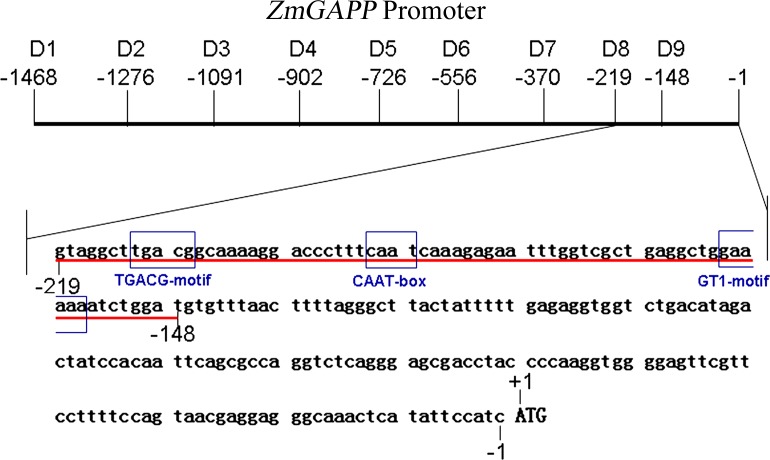
Diagrams of the D8 fragment of the *ZmGAPP* promoter and the 71-bp region for NaCl/PEG stress response. Putative *cis*-regulatory elements in the 71-bp (–219 to –148 bp) sequence of the *ZmGAPP* promoter predicted by PlantCARE and PLACE are shown in the border. CAAT box: common *cis*-acting element in promoter and enhancer regions; TGACG motif: *cis*-acting element involved in MeJA-responsiveness; GT-1 motif: *cis*-acting element involved in pathogen- and NaCl-induced gene expression of *SCaM-4* in soybean and *Arabidopsis*.

In the study, we identified and characterized a salinity or osmotic stress inducible promoter from maize Type-II H^+^-pyrophosphatase gene (*ZmGAPP*) in transgenic tobacco. By analyzing nine 5′ deleted mutants under normal and NaCl or PEG stress conditions, a 219-bp fragment (D8) of the *ZmGAPP* promoter was identified and functionally validated. This fragment may provide an efficient means of conferring high levels of inducible transgene expression (Fig 9). The use of alternative plant promoters suitable to the plant′s background and the type of transgenes utilized is essential for the stacking of multiple genes to avoid the homology dependent gene silencing that often occurs in transgenic plants. The novel D8 fragment isolated from monocot maize described in this study could therefore, be of great use in regulating gene expression in salinity or drought tolerance transgenic breeding of dicot crops based on its heterogeneous promoter activity and inducibility.

Furthermore, a 71-bp segment (–219 to –148 bp) of the *ZmGAPP* promoter was identified as the key region for the plant′s salinity and osmotic stress responsiveness (Fig 8), with analysis of GUS expression in transient transformed tobacco leaves revealing that the 71-bp segment was sufficient for the salinity or osmotic stress response.

## Supporting Information

S1 Fig The constructs of the truncated segments of the *ZmGAPP* promoter fused with *GUSA* and an illustration of restriction digestion analysis. A series of 5′ deleted fragments of the *ZmGAPP* promoter were ligated into the upstream of the *GUSA* gene of the pCAMBIA1391Z vector (A). The numbers indicate the nucleotide position from the translational initiate codon ATG (A as +1). The fused plasmids were confirmed by restriction digestion analysis with HindШ/EcoR1 (B).
(TIF)

S2 Fig PCR analysis and GUS histochemical staining of transgenic tobacco. (A) Genomic PCR analysis of transformed plants using primers HPTFR (Table 1) designed for the hygromycin gene. DL2000 was the marker; + = the PCR result of pCAMBIA1391Z plasmid; CK = non-transformed plants; 1–11 = transformed tobacco plants. (B) GUS histochemical staining of transgenic plants. 35S = transgenic tobacco of the *CaMV 35S* promoter; D1–D9 = transgenic tobacco containing one of nine truncated promoter fragments.
(TIF)

S3 Fig GUS histochemical staining of tobacco leaves in transient assays with different constructs. The *CaMV 35S* represents full-length 35S promoter-driven GUS expression; p-mini35S represents the mini35S (–46 to +10 bp) promoter-driven GUS expression. The test construct p-71bp-mini35S, in which the 71-bp region (–219 to –148 bp) identified in the *ZmGAPP* promoter was fused to the p-mini35S promoter to drive the GUS expression. (A) GUS staining resulting from non-transformed tobacco leaves (WT) and the transient transformed tobacco leaves with constructs *CaMV 35S*, p-mini35S and p-71bp-mini35S under both normal and 200 mM NaCl-stress conditions for 24 h. (B) Histochemical GUS staining resulting from non-transformed tobacco leaves (WT) and the transient transformed tobacco leaves with constructs *CaMV 35S*, p-mini35S and p-71bp-mini35S under both normal and 18% (w/v) PEG 6000-stress conditions for 24 h.
(TIF)
