# Supplementary figures and images for: Isolation and Functional Validation of Salinity and Osmotic Stress Inducible Promoter from the Maize Type-II H+-Pyrophosphatase Gene by Deletion Analysis in Transgenic Tobacco Plants
Source: PLoS One. 2016 Apr 21;11(4):e0154041. doi: 10.1371/journal.pone.0154041 (PMC4839719; doi:10.1371/journal.pone.0154041)

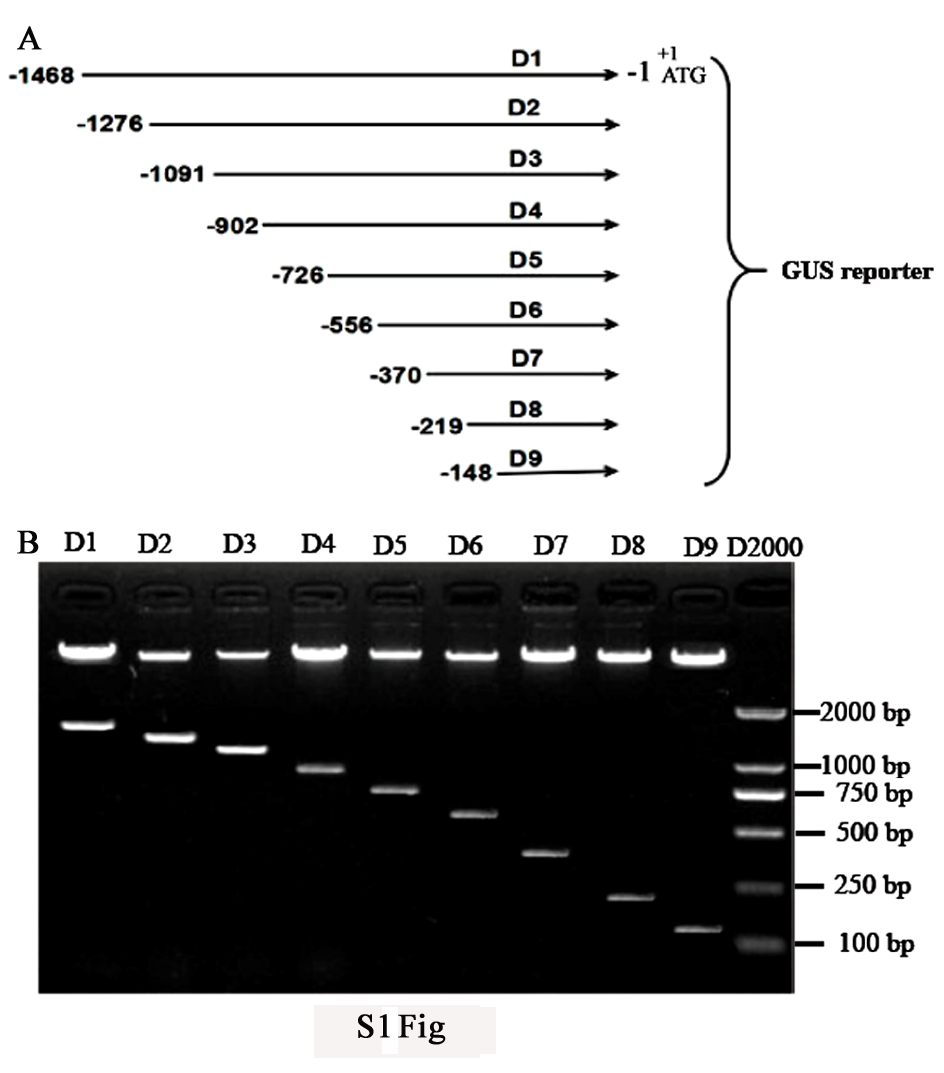

Supplement: S1 Fig — A series of 5′ deleted fragments of the ZmGAPP promoter were ligated into the upstream of the GUSA gene of the pCAMBIA1391Z vector (A). The numbers indicate the nucleotide position from the translational initiate codon ATG (A as +1). The fused plasmids were confirmed by restriction digestion analysis with HindШ/EcoR1 (B). (TIF) [file pone.0154041.s001.tif]

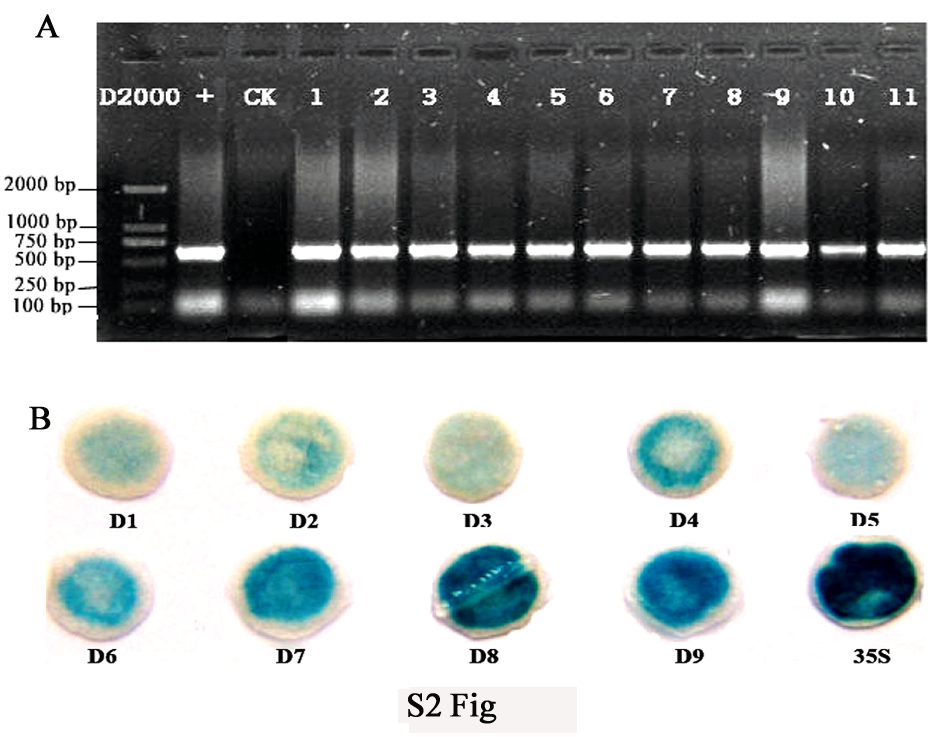

Supplement: S2 Fig — (A) Genomic PCR analysis of transformed plants using primers HPTFR (Table 1) designed for the hygromycin gene. DL2000 was the marker; + = the PCR result of pCAMBIA1391Z plasmid; CK = non-transformed plants; 1–11 = transformed tobacco plants. (B) GUS histochemical staining of transgenic plants. 35S = transgenic tobacco of the CaMV 35S promoter; D1–D9 = transgenic tobacco containing one of nine truncated promoter fragments. (TIF) [file pone.0154041.s002.tif]

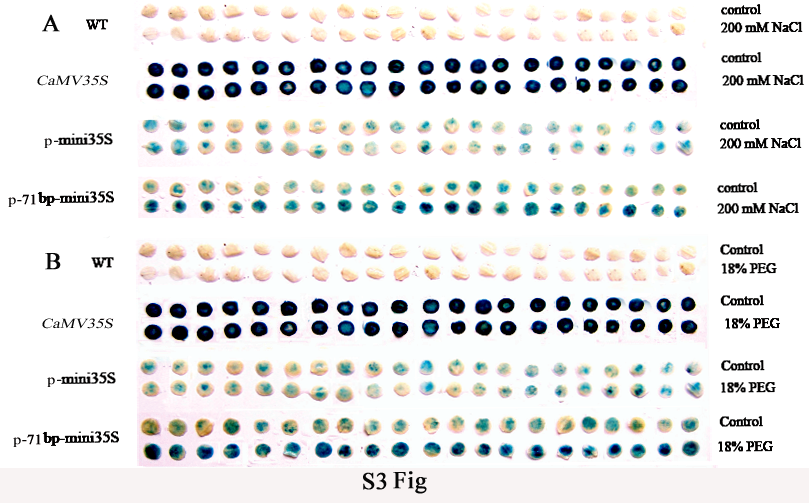

Supplement: S3 Fig — The CaMV 35S represents full-length 35S promoter-driven GUS expression; p-mini35S represents the mini35S (–46 to +10 bp) promoter-driven GUS expression. The test construct p-71bp-mini35S, in which the 71-bp region (–219 to –148 bp) identified in the ZmGAPP promoter was fused to the p-mini35S promoter to drive the GUS expression. (A) GUS staining resulting from non-transformed tobacco leaves (WT) and the transient transformed tobacco leaves with constructs CaMV 35S, p-mini35S and p-71bp-mini35S under both normal and 200 mM NaCl-stress conditions for 24 h. (B) Histochemical GUS staining resulting from non-transformed tobacco leaves (WT) and the transient transformed tobacco leaves with constructs CaMV 35S, p-mini35S and p-71bp-mini35S under both normal and 18% (w/v) PEG 6000-stress conditions for 24 h. (TIF) [file pone.0154041.s003.tif]
